# Curved Planar Reformation: A Useful Method for Screening Dental Pathologies in Chronic Rhinosinusitis via Paranasal Sinus Computed Tomography

**DOI:** 10.3390/tomography8050194

**Published:** 2022-09-16

**Authors:** Wei-Chih Chen, Lisa Alice Hwang, Wei-Che Lin, Ching-Nung Wu, Wei-Chia Su, Kuan-Chung Fang, Sheng-Dean Luo

**Affiliations:** 1 Department of Otolaryngology, College of Medicine, Kaohsiung Chang Gung Memorial Hospital and Chang Gung University, Kaohsiung 833, Taiwan; 2 Department of Oral and Maxillofacial Surgery, College of Medicine, Chiayi Chang Gung Memorial Hospital and Chang Gung University, Chiayi 613, Taiwan; 3 Department of Diagnostic Radiology, College of Medicine, Kaohsiung Chang Gung Memorial Hospital and Chang Gung University, Kaohsiung 833, Taiwan; 4 Department of Oral and Maxillofacial Surgery, College of Medicine, Kaohsiung Chang Gung Memorial Hospital and Chang Gung University, Kaohsiung 833, Taiwan

**Keywords:** curved planar reformations, odontogenic sinusitis, cone-beam computed tomography, dental pathology, periapical lesion, fistula

## Abstract

(1) Background: Curved planar reformation (CPR) is a multiplanar reformatting technique of computed tomography (CT) commonly used during dental cone-beam CT (CBCT) to generate panorex-like images for dental evaluation. Here, we evaluated the utility of an additional CPR sequence in detecting dental pathologies in patients with chronic rhinosinusitis (CRS). (2) Methods: CRS patients who underwent paranasal sinus CT were enrolled retrospectively. The CT images featured three orthogonal sequences and a reconstructed CPR sequence. Additional dental CBCT was performed in patients with pathologies with a strongly suspected odontogenic origin. Dental pathologies detected by CT, CPR, and CBCT were analyzed. (3) Results: A total of 82 CRS patients (37 females and 45 males; mean age 47.3 ± 13.7 years) were included, of whom 23 underwent dental CBCT. In total, 1058 maxillary teeth were evaluated. Compared with paranasal sinus CT, CPR identified greater frequencies of dental pathologies, particularly caries (*p* < 0.001), periapical lesions (*p* < 0.001), and fistulae (*p* = 0.014). CBCT identified greater frequencies of periodontal dental pathologies (*p* = 0.046) and premolar caries (*p* = 0.002) compared with CPR. CBCT and CPR detected molar dental pathologies at similar frequencies. (4) Conclusions: CPR could increase the diagnostic rate of odontogenic pathologies compared with standard CT orthogonal views, especially when the sinusitis is caused by caries, periapical lesions, or fistulae. The addition of a CPR sequence allows for simple screening of dental pathologies in CRS patients without a need for additional radiation.

## 1. Introduction

Chronic rhinosinusitis (CRS) is a highly prevalent inflammatory disease in the upper airway [1]. The estimated prevalence of CRS in Europe and the United States ranges from 10.9% to 12.1% [1,2], and a total of 89,495 sinus surgeries were performed in England between 2010 and 2019 [3]. CRS is a multi-factorial disease caused by a combination of various host and environmental factors; CRS, with an odontogenic source, is considered curable and may need to be treated before sinus surgery. An association between dental infection and sinusitis was first recognized in the 17th century when the anatomist and physician Nathaniel Highmore identified an abscess within a canine tooth as the cause of a maxillary sinus infection [4]. Today, however, odontogenic causes of rhinosinusitis are often overlooked. Of all chronic maxillary sinusitis cases, 10–25% are of an odontogenic origin, although this rate may reach 70–75% among patients with unilateral sinusitis [5,6,7,8]. Such high variability may be attributable to diagnostic difficulties, such as pathologies going unnoticed on the initial examination. The symptoms of odontogenic sinusitis are indistinguishable from non-odontogenic sinusitis, and specific symptoms such as tooth pain are only present in 30% of odontogenic sinusitis patients [7,9]. Previous studies found that many odontogenic sinusitis pathologies were not detected by initial computed tomography (CT) evaluations [5,10].

CT can provide three-dimensional (3D) volumetric datasets of the anatomic areas, which contain both objects of interest and less interest. The concept of curved planar reformation (CPR) is to display specific objects of interest within a single image for review. CPR is a kind of multiplanar reformatting technique used to visualize specific anatomical structures. A centerline of interested structures is drawn on the planar image of three-dimensional volumetric datasets acquired by spiral CT. CPR is then generated by re-sampling and visualizing the 3D volumetric information gained from the centerline detection process. If an anatomical structure is curved or tubular, CPR can simultaneously show important details better than traditional planar cross-sections. CPR has been used to evaluate vascular abnormalities [11], spinal disease [12], the biliary tract [13], and the urinary tract [14]. A CPR follows a curved path to display the entire course of the dental arch by generating a reconstructed thin-slice panoramic image. This technique is commonly used by dentists during dental cone-beam computed tomography (CBCT) to scan for dentoalveolar pathologies and to plan surgery [15,16,17].

Conventional paranasal CT has been considered the gold standard for the evaluation of paranasal sinus diseases; however, dental pathologies may thus be underdiagnosed. Thus, adding CPR to the routine axial, coronal, and parasagittal CT views may have a benefit. The panorex-like CPR views could help clinicians or radiologists simultaneously evaluate dental pathologies of maxillary teeth. We assume that CPRs may aid the screening of dental pathologies in patients with chronic rhinosinusitis (CRS). Here, we explore whether CPRs are better than regular paranasal sinus CT views in revealing dental pathologies in patients with CRS.

## 2. Materials and Methods

### 2.1. Study Design

We retrospectively analyzed paranasal sinus CT data obtained from Kaohsiung Chang Gung Memorial Hospital. Patients with CRS who had received paranasal sinus CT (Lund–Mackay score ≥ 1) between January 2016 and April 2017 were enrolled. Their clinicopathological characteristics were obtained from clinical records, including age, sex, and sinonasal endoscopic findings. The objective disease severity was evaluated by CT images with the Lund–Mackay scoring system [18] and endoscopic images with the modified Lund–Kennedy scoring system [19]. Exclusion criteria included a history of head and neck cancer, prior irradiation of the head and neck region, and toothlessness. The study was approved by the medical ethical and human clinical trial committees of Chang Gung Memorial Hospital (ref. 201901320B0). The need for informed patient consent was waived given the retrospective nature of the work. 

**Figure 1:**
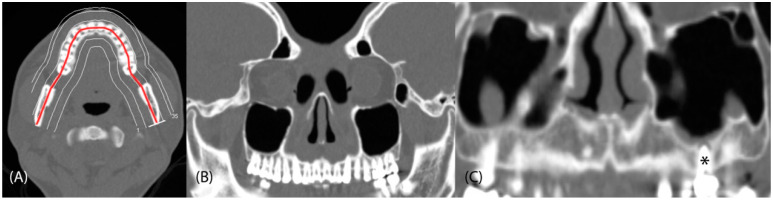
Continuous panoramic images of the maxillary teeth for detecting dental pathologies were yielded after CPR. (**A**) A series of CPRs were generated by drawing a reference line (red line) along the centerline corresponding to the maxillary teeth. Continuous CPR images were reconstructed according to lines parallel to the reference line. (**B**) A simulated dental panoramic image was created after reconstruction. (**C**) CPR detected a periapical abscess with a fistula in a left maxillary molar (no. 26, *).

**Figure 2:**
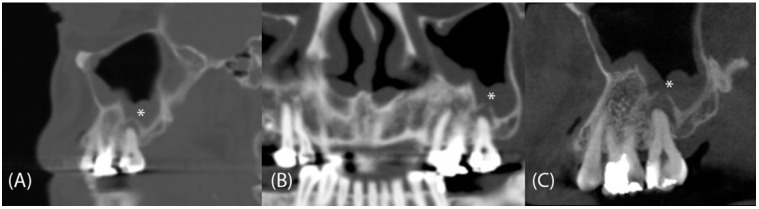
CPR and dental CBCT could identify more dental pathologies than conventional CT images. (**A**) The parasagittal CT view identified a periapical lesion of a left maxillary molar (no. 27, asterisk); however, both (**B**) CPR and (**C**) CBCT revealed a co-existing fistula (*).

Paranasal sinus CT was performed using a multislice CT scanner (Siemens Somatom Definition Flash AS 64 slice, Munich, Germany) with a protocol of 40 mAs, 120 kV, and 0.6 mm. All images were prospectively reconstructed at 0.75 mm with a 0.4 mm overlap using a soft-tissue filter and high-resolution bone filter. The CT images were presented as three orthogonal sequences (axial, coronal, and parasagittal views). CPR images were prepared on a workstation (Siemens Healthineers syngo^®^ Multimodality Workplace, Erlangen, Germany) using the cursor to draw curved lines on the axial views of the maxillary dental fossae. This process was completed by the built-in program and usually needs an additional 3 to 5 min. This yielded continuous panoramic images of the maxillary teeth with a thickness of 1 mm (Figure 1). Patients whose clinical examinations did not rule out odontogenic disease also underwent dental CBCT (slice thickness 0.3 mm); we compared the paranasal sinus CT and dental CBCT data (Figure 2). 

### 2.2. Assessment of Dental Pathologies and Sinus Conditions

The dental pathologies and their locations in routine CT orthogonal views, as well as CPRs, were independently recorded by two oral and maxillofacial surgeons (Dr. Su and Dr. Hwang). Any inter-examiner disagreement was resolved by consensus. The maxillary teeth were divided into the anterior teeth (incisors and canines), premolars, and molars. Odontogenic pathologies including caries, periapical lesions, periodontal lesions, fractures, fistulae, and dental implants were evaluated and recorded. Caries were diagnosed when a radiolucent region was noted over the occlusal and proximal sides of a tooth featuring a clear enamel perforation. A periapical lesion was diagnosed when an apical radiolucency of the root exceeded twice the width of the periodontal ligament space [20]. A periodontal lesion was diagnosed when the bone height was 3 mm below the cementoenamel junction with the apex [21]. A fracture was diagnosed when linear radiolucency was evident across a tooth with discontinuity in its dental structure. A fistula was diagnosed when an oroantral communication was noted. 

### 2.3. Statistical Analysis

Statistical analysis was performed using SPSS version 22 software (IBM Corp., Armonk, NY, USA). A *p*-value less than 0.05 was considered as indicating statistical significance. The Wilcoxon signed-rank test was used to compare the dental pathologies revealed by routine CT sequences versus CPR and CPR versus CBCT.

## 3. Results

We included 82 CRS patients (37 females and 45 males) aged 15–85 years (mean age 47.3 ± 13.7 years). All patients underwent non-contrast paranasal sinus CT (axial, coronal, parasagittal, and CPR sequences), and 23 patients further received dental CBCT. Bilateral CRS was found in 36 (43.9%) patients, and CRS with nasal polyp was found in 27 (32.9%) patients. The Lund–Mackay CT scores and modified Lund–Kennedy endoscopic scores were 7.3 ± 5.9 and 3.2 ± 2.5, respectively. The clinical characteristics are listed in Table 1. In total, 1058 maxillary teeth were evaluated. The prevalence of dental pathologies diagnosed by CT and CPR were 12.9% and 20.5%, respectively. Approximately half of all pathologies were located in the molars. We recorded 136 pathologies (24 caries, 53 periapical lesions, 39 periodontal lesions, 2 fractures, 9 fistulae, and 9 implants) on conventional paranasal sinus CT orthogonal views and 217 pathologies (61 caries, 89 periapical lesions, 40 periodontal lesions, 3 fractures, 15 fistulae, and 9 implants) on CPR sequences. The numbers of identified dental pathologies significantly differed between paranasal sinus CT and CPR for caries (*p* < 0.001), periapical lesions (*p* < 0.001), and fistulae (*p* = 0.014). CPR afforded higher diagnostic rates for the anterior teeth, premolars, and molars compared with CT (Table 2).

In total, 311 maxillary teeth in 23 patients were evaluated by both dental CBCT and CPR. In these patients, 96 dental pathologies (28 caries, 27 periapical lesions, 31 periodontal lesions, 1 fracture, and 9 fistulae) were identified by CBCT and 72 (17 caries, 27 periapical lesions, 16 periodontal lesions, 2 fractures, and 10 fistulae) by CPR. The number of dental pathologies identified differed between CBCT and CPR for periodontal lesions (*p* = 0.046), periapical lesions in the anterior teeth (*p* = 0.046), and caries in the premolars (*p* = 0.002). The diagnostic rate of premolar pathologies was better for CBCT than CPR, but both modalities effectively diagnosed anterior teeth and molar pathologies (Table 3).

## 4. Discussion

This study demonstrated that CPR detected more odontogenic pathologies in incisors, canines, premolars, and molars than conventional orthogonal views. The CPR sequence was more sensitive in detecting caries, periapical lesions, and fistulae, particularly in molars, which are at high risk for odontogenic sinusitis [22]. However, the various modalities were of equal sensitivity in detecting periodontal lesions and implants. Thus, CPRs reconstructed from paranasal sinus CT data are superior to those of the three standard orthogonal planes (axial, sagittal, and coronal) when evaluating dental pathologies. The CPR sequence yields thin panorex-like images that facilitate the simultaneous evaluation of dental conditions. We consider that the addition of a CPR sequence when evaluating CRS patients increases the diagnostic rate of synchronous odontogenic pathologies. In addition, CPR evaluations are simple and could become routine in the clinic.

The close anatomical association between the maxillary teeth and maxillary sinuses promotes the transmission of periapical or periodontal infections into the maxillary sinus. In a series of 200 maxillary sinuses visible on CBCT examinations, the periapical lesions, periodontal bone loss, severe caries, and extracted teeth significantly increased maxillary sinus mucosal thickening. On the contrary, the distance between root apices and the maxillary sinus floor negatively correlated with the thickness of sinus mucosa [23]. Lechien et al. reported the most commonly involved maxillary teeth are the first molars (35.6%), followed by the second (22%) and third molars (17.4%), although incisor and canine infections have also been reported as causes of odontogenic sinusitis [22]. In their study, periapical pathologies accounted for 25.1% of cases. Our present CPR series showed similar results, with 52.8% of all pathologies involving the molars and 37.5% of all pathologies being periapical lesions.

Odontogenic sinusitis should be considered when evaluating patients with rhinosinusitis. Due to the awareness of dental health and occlusion’s function, the need for dental procedures and implants has increased in recent years [24]. Otherwise, the incidence of iatrogenic odontogenic sinusitis has also increased [25]. However, current diagnostic criteria for odontogenic sinusitis that were used in previous studies are extremely heterogeneous [26]. The temporal relationship between dental problems and symptoms of rhinosinusitis would raise suspicions of odontogenic origins. The most common initial symptoms of odontogenic sinusitis were facial pain, postnasal discharge, and nasal congestion. These symptoms are nonspecific and would not help in the diagnosis of odontogenic sinusitis [27]. Although odontogenic sinusitis usually presents as unilateral maxillary rhinosinusitis, Matsumoto et al. found odontogenic infections in 45.3% of bilateral rhinosinusitis patients [28]. Therefore, screening for a possible odontogenic focus in CRS is extremely important. Currently, CBCT serves as the standard diagnostic method for dental pathologies. Dental CBCT affords a high spatial resolution and accurate detection of periapical lesions, oroantral fistulae, and periodontal diseases, especially in upper maxillary teeth [15,29,30]. We found that CPR had an acceptable sensitivity in detecting pathologies in the molars, which are the teeth most commonly involved in odontogenic sinusitis. The diagnostic rates of periapical lesions and fistulae by CPR and dental CBCT were comparable, but periodontal lesions were diagnosed more frequently by CBCT. In summary, the diagnostic rate of CPR is slightly inferior to CBCT. However, the CPR series is reconstructed from prior paranasal sinus CT data, eliminating the need for additional radiation. Otherwise, CPR is presented as a panoramic series of maxillary teeth which facilitates the clinician comprehensively evaluating the dental condition. Therefore, CPR could be a screening tool before sinus surgery.

The pathophysiology, microbiology, and management of odontogenic sinusitis differ from those of non-odontogenic sinusitis; accurate diagnosis enhances treatment success [31]. Most authors agreed that treatment of the underlying dental pathology should be the first step in the treatment of odontogenic sinusitis [32]. Yoo et al. reported that two-thirds of patients with odontogenic sinusitis caused by dental caries or periapical abscesses could be cured by medical and dental treatment without a need for functional endoscopic sinus surgery (FESS) [33]. Simuntis et al. reported that 22.9% of patients had persistent symptoms after eliminating dental causes and needed further sinus surgery [34]. An unrecognized dental pathology may be associated with recalcitrant sinusitis after FESS [35]. The early detection of underlying dental pathologies is very important for CRS patients. However, odontogenic sinusitis can be easily missed [5]. Dental pathologies are not always detected initially; 35–70% of sinus CT reports on odontogenic sinusitis patients lacked any mention of dental pathologies [5,10]. The radiology community does not understand the importance of examining teeth when reviewing sinus CT data. Odontogenic conditions that cause rhinosinusitis are often overlooked by radiologists, dentists, and even otolaryngologists [36].

Currently, paranasal sinus CT is considered the gold standard for the diagnosis of rhinosinusitis; however, this modality may not always reveal an odontogenic cause. To the best of our knowledge, this is the first study to assess whether CPR could become a standard protocol to detect potential odontogenic origins in CRS patients. Odontogenic sinusitis was classified as a specific phenotype of secondary CRS with localized disease in the 2020 European Position Paper on Rhinosinusitis, which stated that odontogenic sources should be adequately treated before FESS [37]. Therefore, we used CPR to screen for possible dental pathologies in CRS patients. The limitations of the present study include its retrospective design and the small number of patients who received dental CBCT after dental evaluation by a dentist. In addition, we lack definitive proof that the rhinosinusitis cases were related to dental infections. Further prospective studies incorporating CPR into CRS management protocols in clinical practice should be performed.

## 5. Conclusions

Odontogenic sinusitis, categorized as secondary CRS, emphasized the importance of addressing and treating dental pathologies before sinus surgery. The CPRs facilitated clinicians to evaluate dental conditions simultaneously and increased the diagnostic rate of dental disease without exposing the patient to additional radiation. In clinical practice, the addition of CPR could be a routine CT protocol for screening dental status in CRS patients. The CPR images accompanied by sinus CT could facilitate reviews before sinus surgery to identify the possible odontogenic causes. The patients with dental pathologies would be referred to the dentist for further dental evaluation and management. The sinus condition of CRS patients with dental pathologies would be re-evaluated after adequate dental treatment. This protocol could increase the diagnostic rate of odontogenic sinusitis and avoid unnecessary sinus surgeries.

## Figures and Tables

**Table 1:** Baseline characteristics of these 82 CRS patients.

Patient Characteristics	Mean ± SD	n (%)
Age (years)	47.3 ± 13.7	
Sex		
Male (%)		45 (54.9%)
Female (%)		37 (45.1%)
CRS side		
Right (%)		25 (30.5%)
Left (%)		21 (25.6%)
Bilateral (%)		36 (43.9%)
CRS with nasal polyp		27 (32.9%)
Lund–Mackay CT score	7.3 ± 5.9	
Modified Lund–Kennedy endoscopic score	3.2 ± 2.5	
Dental CBCT		23 (28.1%)

CRS = Chrnoic rhinosinusitis; CT = computed tomography; CBCT = cone-beam computed tomography; SD = standard deviation.

**Table 2:** Comparison of dental pathologies diagnosed by paranasal sinus CT and CPR in 1058 maxillary teeth.

Dental Pathology	Paranasal Sinus CT, n (%)	CPR, n (%)	*p*
All pathologies	136 (12.9%)	217 (20.5%)	<0.001 *
Molars	75 (55.1%)	114 (52.5%)	<0.001 *
Premolars	29 (21.3%)	52 (24.0%)	<0.001 *
Anterior teeth	32 (23.5%)	51 (23.5%)	0.001 *
Periapical lesions	53 (39.0%)	89 (41%)	<0.001 *
Molars	17	26	0.029 *
Premolars	15	30	0.004 *
Anterior teeth	21	33	0.002 *
Caries	24 (17.6%)	61 (28.1%)	<0.001 *
Molar	16	41	0.001 *
Premolar	5	12	0.020 *
Anterior teeth	3	8	0.059
Periodontal lesions	39 (28.7%)	40 (18.4%)	0.317
Molars	28	28	1.000
Premolars	4	4	1.000
Anterior teeth	7	8	0.317
Fistulae	9 (6.6%)	15 (6.9%)	0.014 *
Molars	9	14	0.025 *
Premolars	0	1	0.317
Anterior teeth	0	0	1.000
Fractures	2 (1.5%)	3 (1.4%)	0.317
Molars	1	1	1.000
Premolars	1	1	1.000
Anterior teeth	0	1	0.317
Implants	9 (6.6%)	9 (4.1%)	1.000
Molars	4	4	1.000
Premolars	4	4	1.000
Anterior teeth	1	1	1.000

* *p* < 0.05; CT = computed tomography; CPR = curved planar reformation.

**Table 3:** Comparison of dental pathologies diagnosed by CBCT and CPR in 331 maxillary teeth.

Dental Pathology	Dental CBCT, n (%)	CPR, n (%)	*p*
All pathologies	96 (30.9%)	72 (23.2%)	0.018 *
Molars	46 (47.9%)	38 (52.8%)	0.303
Premolars	37 (38.5%)	23 (31.9%)	0.008 *
Anterior teeth	13 (13.5%)	11 (15.3%)	0.577
Periapical lesions	27 (28.1%)	27 (37.5%)	1.000
Molars	11	13	0.414
Premolars	8	10	0.414
Anterior teeth	8	4	0.046 *
Caries	28 (29.2%)	17 (23.6%)	0.140
Molar	7	6	0.915
Premolar	19	8	0.002 *
Anterior teeth	2	3	0.705
Periodontal lesions	31 (32.3%)	16 (22.2%)	
Molars	20	10	0.161
Premolars	8	3	0.059
Anterior teeth	3	3	1.000
Fistulae	9 (9.4%)	10 (13.9%)	0.705
Molars	8	9	0.564
Premolars	1	1	1.000
Anterior teeth	0	0	1.000
Fractures	1 (1.0%)	2 (2.8%)	0.317
Molars	0	0	1.000
Premolars	1	1	1.000
Anterior teeth	0	1	0.317

* *p* < 0.05; CBCT = Cone-beam computed tomography; CPR = Curved planar reformation.

## Data Availability

Not applicable.
